# Supplementary material for: The anterior insular cortex unilaterally controls feeding in response to aversive visceral stimuli in mice
Source: Nat Commun. 2020 Jan 31;11:640. doi: 10.1038/s41467-020-14281-5 (PMC6994462; doi:10.1038/s41467-020-14281-5)
Supplement: Supplementary file 1 — Supplementary Information [file 41467_2020_14281_MOESM1_ESM.pdf]

Supplementary information

The anterior insular cortex unilaterally controls feeding in responses to aversive visceral stimuli in mice

Wu et al.

a

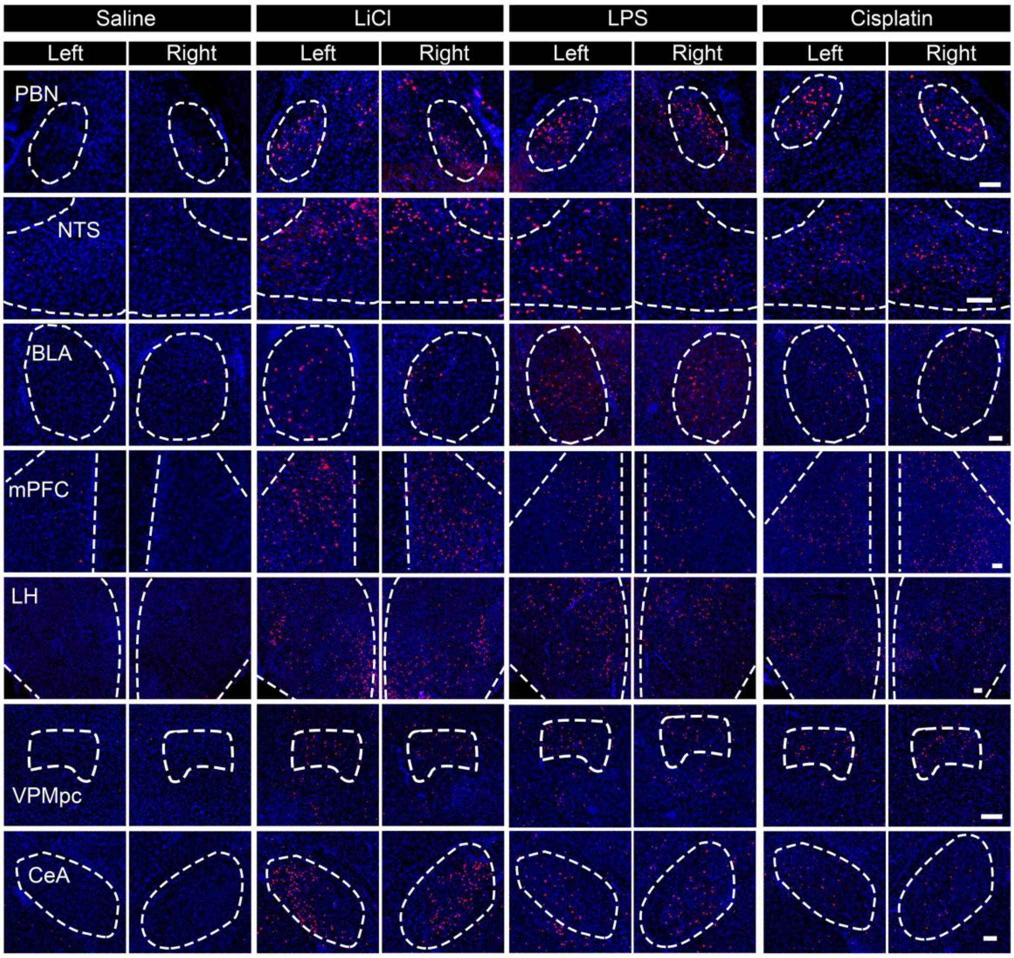

b

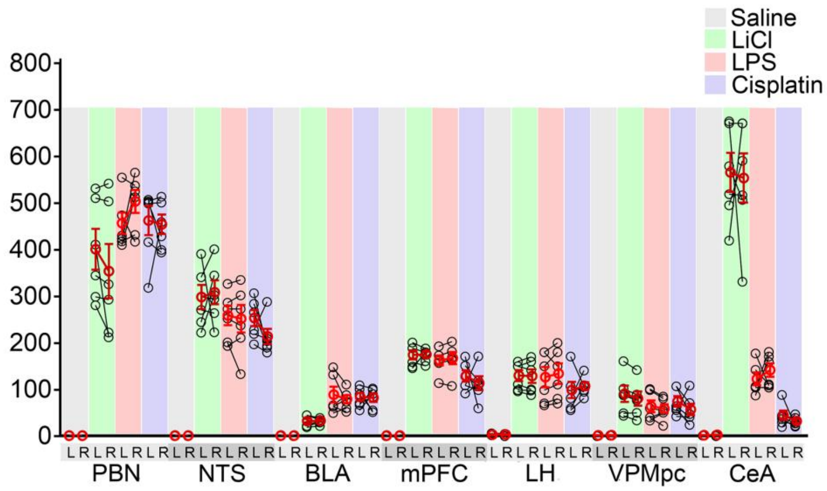

Supplementary Figure 1. Activation of different brain regions by aversive stimuli.

(a,b) Representative histology (a) and quantification (b) of Fos-like immunoreactivity in the left and right PBN, NTS, BLA, LH, CeA, VPMpc, and mPFC after intraperitoneal injection of Saline, LiCl (150 mg per kg), LPS (0.1 mg per kg) or Cisplatin (4 mg pre kg). PBN,

parabrachial nucleus; NTS, nucleus of the solitary tract; BLA, basolateral amygdaloid nucleus; LH, lateral hypothalamus; CeA, central amygdala; VPMpc, the parvicellular part of the ventroposteromedial nucleus of the thalamus, mPFC, medial prefrontal cortex (n = 6 mice per group, Two-way ANOVA with Bonferroni *post hoc* analysis). Scale bar, 100 μm. Source data are provided as a Source Data file.

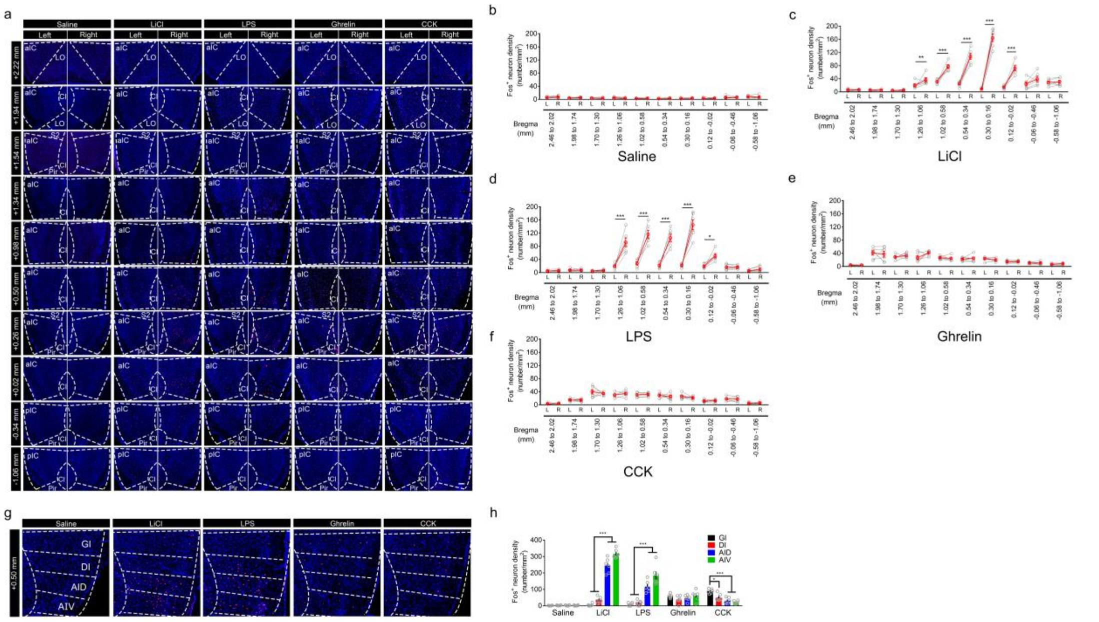

Supplementary Figure 2. Activation of different subregions of the IC by different stimuli. (a-f) Representative histology (a) and quantification (b-f) of Fos-like immunoreactivity in the left or right IC from different Bregma after intraperitoneal injection of Saline, LiCl, LPS, Ghrelin or CCK (n = 6 mice per group, Two-way ANOVA with Bonferroni *post hoc* analysis). (g-h) Representative histology (g) and quantification (h) of Fos-like immunoreactivity in the different part of the caudal segment of the right aIC after intraperitoneal injection of Saline, LiCl, LPS, Ghrelin or CCK (n = 6 mice per group, Two-way ANOVA with Bonferroni *post hoc* analysis). **P* < 0.05; ***P* < 0.01; ****P* < 0.005. Red line represents averaged data. Data are presented as means ± SEM. Source data are provided as a Source Data file.

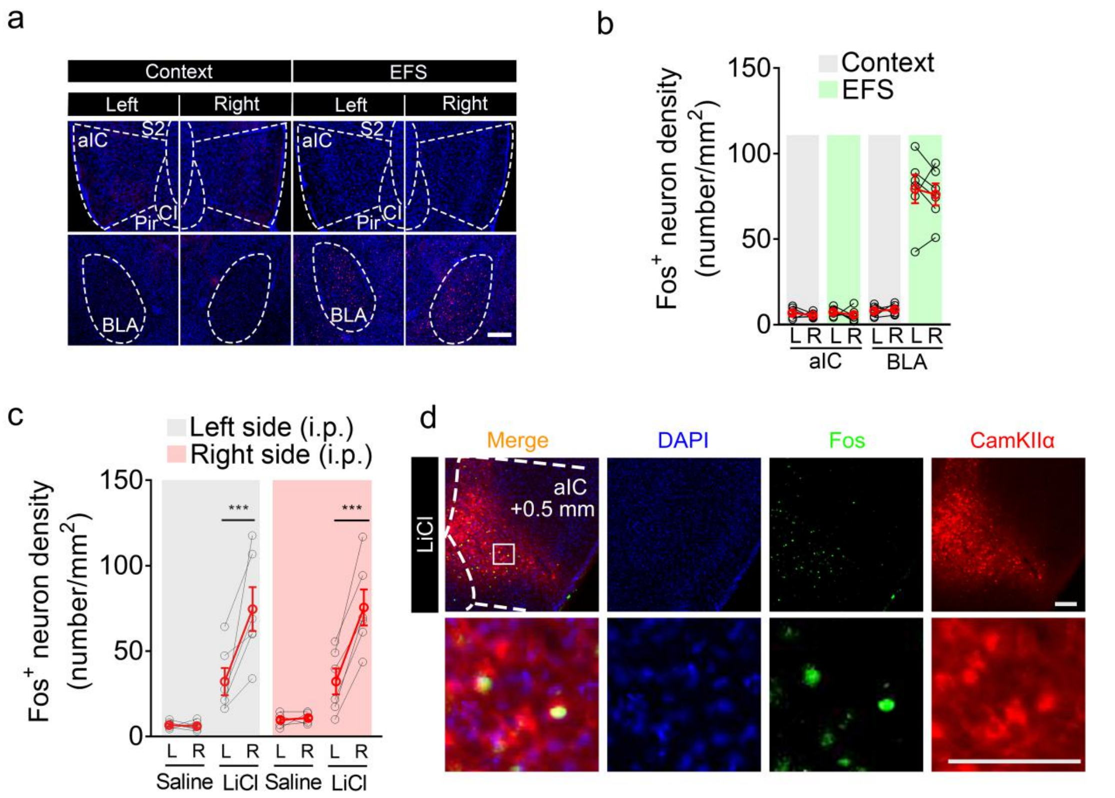

Supplementary Figure 3. Colocalization of LiCl-induced Fos^{+} neurons with CamKII-expressing neuron in the aIC.

(a, b) Representative histology (a) and quantification (b) of Fos-like immunoreactivity in the left and right aIC or BLA after electric shock (EFS, 0.6 mA, 3 times) or contextual control (n = 6 mice per group, Two-way ANOVA with Bonferroni *post hoc* analysis). Scale bar, 100 µm. (c) Statistical analysis of Fos expression in both sides of the aIC after LiCl injection (i.p.) in the left side or right side of the mice body (n = 6 mice per group, Two-way ANOVA with Bonferroni *post hoc* analysis). (d) LiCl (i.p.) treatment-induced Fos expression in the caudal segment of the right aIC of the mice injected with AAV9-CamKII-mCherry (n = 3 mice). **P* < 0.05; ****P* < 0.005. Red line represents averaged data. Data are presented as means ± SEM. Source data are provided as a Source Data file.

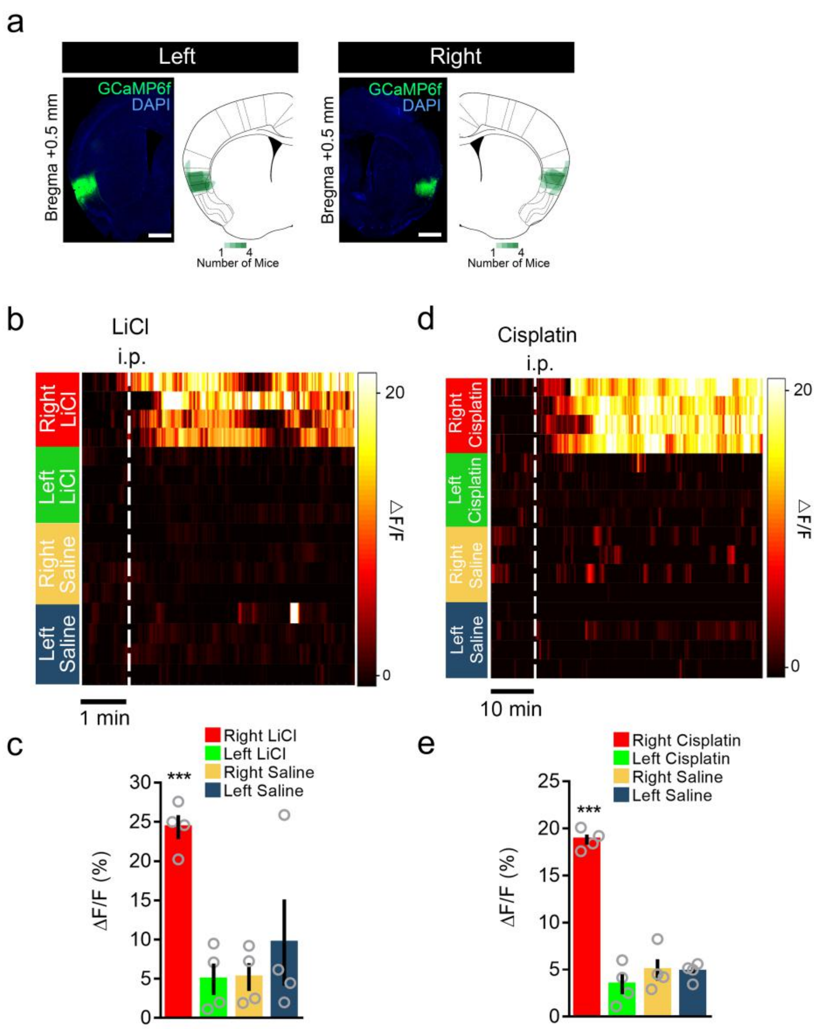

Supplementary Figure 4. Activation of CamKII$^+$ neurons in the caudal segment of the aIC via photometry.

(a) Brain slices from mice with mCamKIIα-GCaMP6f virus injected into the caudal segment of the left or right aIC. Blue, DAPI; green, viral targeting. Dashed black lines are boundaries of subregions. Overlay of mCamKIIα-GCaMP6f expression in 4 mice for each group. Scale bar, 1 mm. (b, c) Heat map of GCaMP6f fluorescence responses of both sides of the aIC^{CamKII} neurons injected with saline or LiCl (b) and quantification of peak Δ*F/F (%)* (c). n = 4 mice per group. (d,e) Heat map of GCaMP6f fluorescence responses of both sides of the aIC^{CamKII} neurons injected with saline or Cisplatin (d) and quantification of peak Δ*F/F* (e). n = 4 mice per group. One-way ANOVA with Bonferroni *post hoc t* test. ***$P < 0.005$. Data are presented as means ± SEM. Source data are provided as a Source Data file.

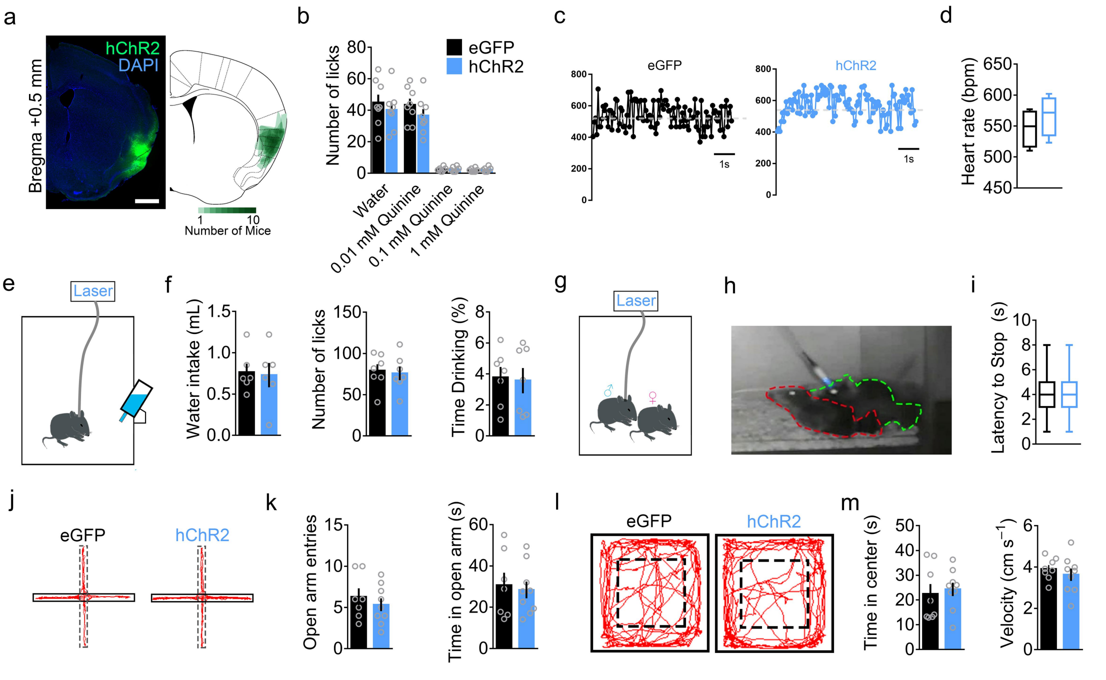

Supplementary Figure 5. Activation of CamKII^{+} neurons in the caudal segment of the right aIC has no effect on drinking, mating or anxiety-like behaviors.

(a) Brain slices from *Camk2a-Cre* transgenic mice with DIO-hChR2(H134R)-eGFP virus injected into the caudal segment of the right aIC. Blue, DAPI; green, viral targeting. Dashed black lines are boundaries of subregions. Overlay of DIO-hChR2(H134R)-eGFP expression in 10 mice. Scale bar, 1 mm. (b) Number of licks of quinine solution with different concentration were tested with 20-Hz photostimulation in hChR2-expressing mice (n = 8 mice per group, Two-way ANOVA with Bonferroni *post hoc* analysis). (c) Representative heart rate for photostimulation (473 nm) in anaesthesia mice. (d) Quantification of heart rate (n = 5 mice per group, Two-tailed unpaired *t* test). (e) Schema to assess water drinking. (f) Total water consumption (left), number of licks (middle) and total drinking time (right) were measured (n = 7 mice per group, Two-tailed unpaired *t* test). (g) Schema to assess mating behavior. (h) Sample video frame showing mating between male *Camk2a-Cre* mice expressing hChR2-eGFP in the right aIC with wild-type female mice. (i) Quantification of latency to stop mating (n = 7 mice per group, Two-tailed unpaired *t* test). (j) Representative locomotor activity traces for photostimulation (473 nm) in elevated plus-maze test assay. (k) Quantification of the number of open arm entries (left) and total time in open arm (right) (n =

7 mice per group, Two-tailed unpaired *t* test). (l) Representative locomotor activity traces for photostimulation (473 nm) in open filed test (OFT) assay. (m) Quantification of total time in center (left) and locomotor activity (right) (n = 7 mice per group, Two-tailed unpaired *t* test). Data are presented as means ± SEM. Source data are provided as a Source Data file.

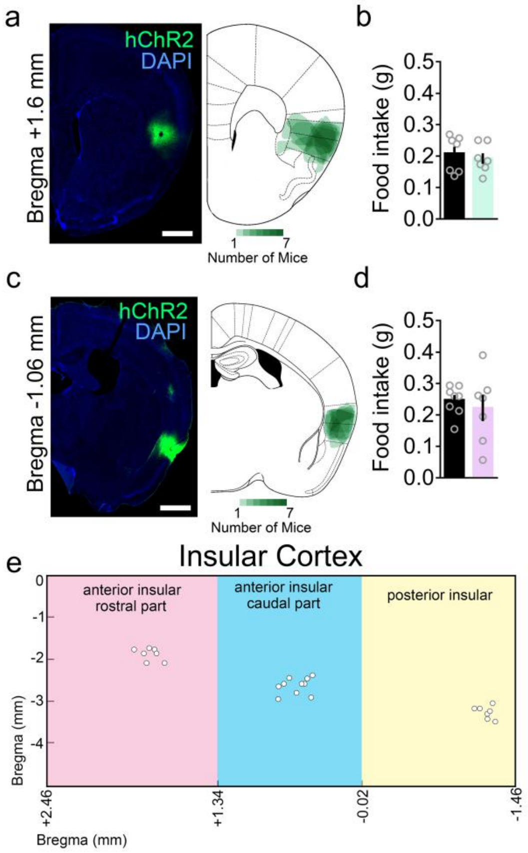

Supplementary Figure 6. Activation of the CamKII⁺ neurons in the rostral segment of the right aIC or in the right pIC has no effect on food consumption in 24-h fasted mice.

(a) Brain slices from *Camk2a-Cre* transgenic mice injected with DIO-hChR2-eGFP into the rostral segment of right aIC. Blue, DAPI; green, viral targeting. Dashed black lines are boundaries of subregions. Overlay of DIO-hChR2-eGFP expression in 7 mice. Scale bar, 1 mm. (b) Total food consumption was measured during a 20-min test, n = 7 for each group. (c) Brain slices from *Camk2a-Cre* transgenic mice injected with DIO-hChR2-eGFP into the right pIC. Blue, DAPI; green, viral targeting. Dashed black lines are boundaries of subregions. Overlay of DIO-hChR2-eGFP expression in 7 mice. Scale bar, 1 mm. (d) Total food consumption was measured during a 20-min test, n = 8 for eGFP mice, n = 7 for hChR2 mice. (e) Summary of the implantation sites of optic fiber in the right insula for photoactivation of the rostral segment of the aIC, the caudal segment of the aIC, and the pIC. Two-tailed unpaired *t* test. Data are presented as means ± SEM. Source data are provided as a Source Data file.

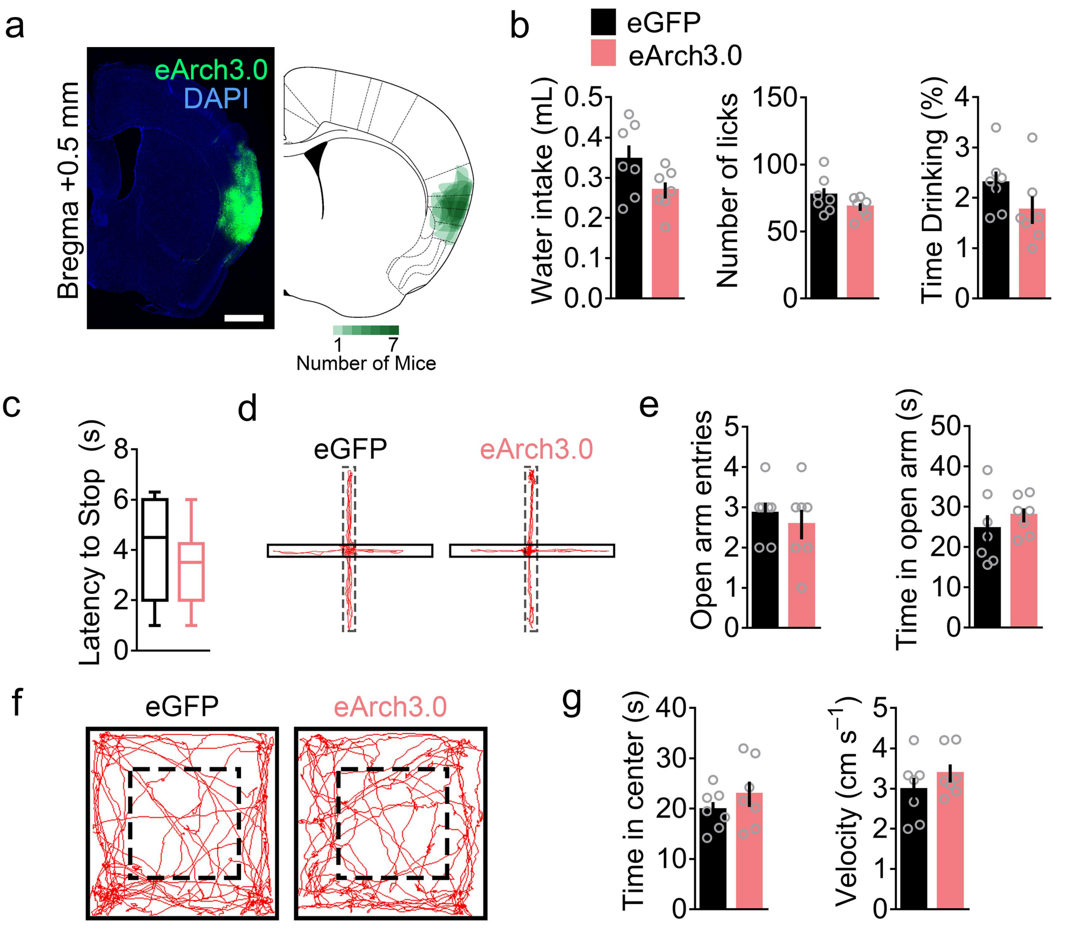

Supplementary Figure 7. Inhibition of the right aIC^{CaMKII} neurons has no effect on drinking, mating or anxiety-like behaviors.

(a) Brain slices from mice with DIO-eArch3.0-eGFP virus injected into the right aIC. Blue, DAPI; green, viral targeting. Dashed black lines are boundaries of subregions. Overlay of DIO-eArch3.0-eGFP expression in 7 mice. Scale bar, 1 mm. (b) Total water consumption (left), number of licks (middle) and total drinking time (right) were measured (n = 7 mice per group). (c) Quantification of latency to stop mating (n = 7 mice per group). (d) Representative locomotor activity traces for photoinhibition (532 nm) in EMPT. (e) Quantification of the number of open arm entries (left) and total time in open arm (right) (n = 7 mice per group). (f) Representative locomotor activity traces for photoinhibition (532 nm) in OFT. (g) Quantification of total time in center (left) and locomotor activity (right) (n = 7 mice per group). Two-tailed unpaired *t* test. Data are presented as means ± SEM. Source data are provided as a Source Data file.

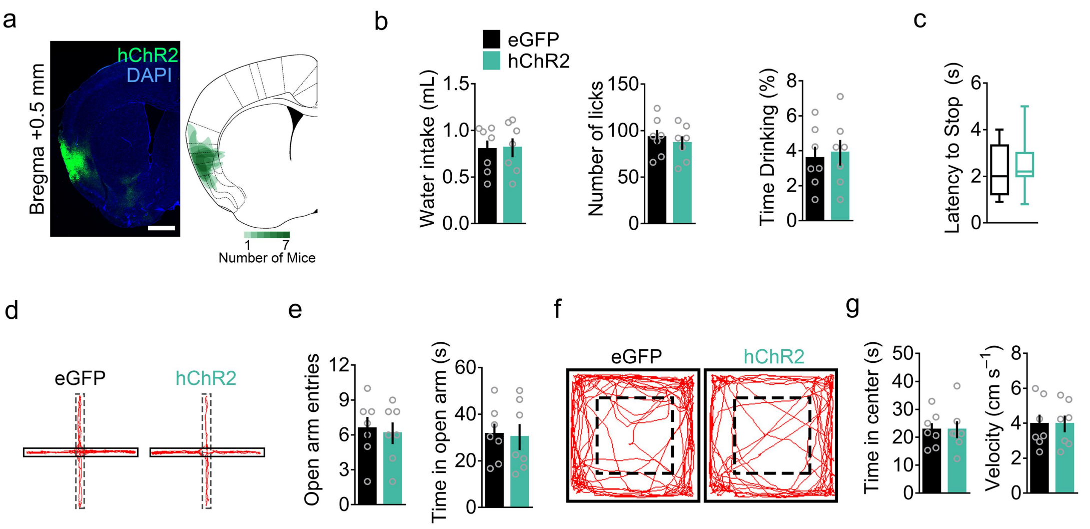

Supplementary Figure 8. Activation of the left aIC^{CaMKII} neurons has no effect on feeding, drinking, mating or anxiety-like behaviors.

(a) Brain slices from mice with hChR2-eGFP virus injected into the left aIC. Blue, DAPI; green, viral targeting. Dashed black lines are boundaries of subregions. Overlay of DIO-hChR2 (H134R)-eGFP expression in 7 mice. Scale bar, 1 mm. (b) Total water consumption (left), number of licks (middle) and total drinking time (right) were measured (n = 7 mice per group). (c) Quantification of latency to stop mating (n = 7 mice per group). (d) Representative locomotor activity traces for photostimulation (473 nm) in EMPT. (e) Quantification of the number of open arm entries (left) and total time in open arm (right) (n = 7 mice per group). (f) Representative locomotor activity traces for photostimulation (473 nm) in OFT. (g) Quantification of total time in center (left) and locomotor activity (right) (n = 7 mice per group). Two-tailed unpaired *t* test. Data are presented as means ± SEM. Source data are provided as a Source Data file.

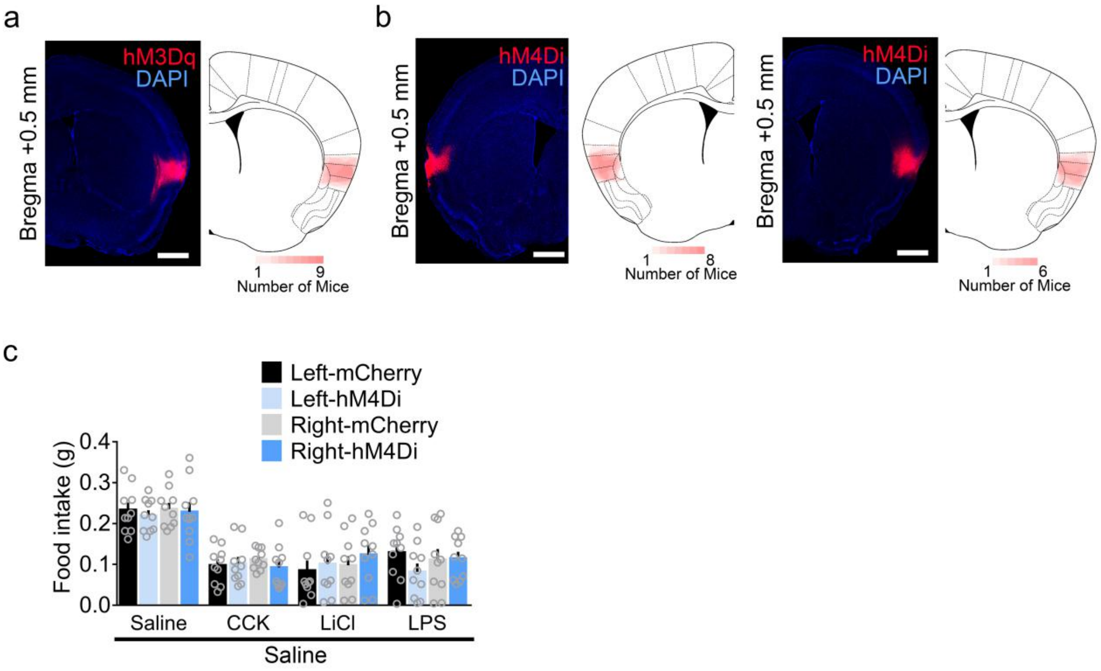

Supplementary Figure 9. Expression of DREADD virus in the injected IC.

(a,b) Brain slices from mice with hM3Dq-DIO-mCherry or hM4Di-DIO-mCherry injected into the aIC. Blue, DAPI; red, viral targeting. Dashed black lines are boundaries of subregions. Overlay of DIO-hM3Dq-mCherry expression in 7 mice. Overlay of DIO-hM4Di-mCherry expression in 8 mice (left aIC) and in 6 mice (right aIC). Scale bar, 1 mm. (c) Food intake in 24-h fasted mice after administration of different anorexigenic agents (CCK (5 µg per kg), LiCl (150 mg per kg) and LPS (0.1 mg per kg)) and saline (n = 8 for left-mCherry, left-hM4Di and right-mCherry group, respectively, n = 6 for right-hM4Di group). Two-way ANOVA with Bonferroni *post hoc* analysis. Data are presented as means ± SEM. Source data are provided as a Source Data file.

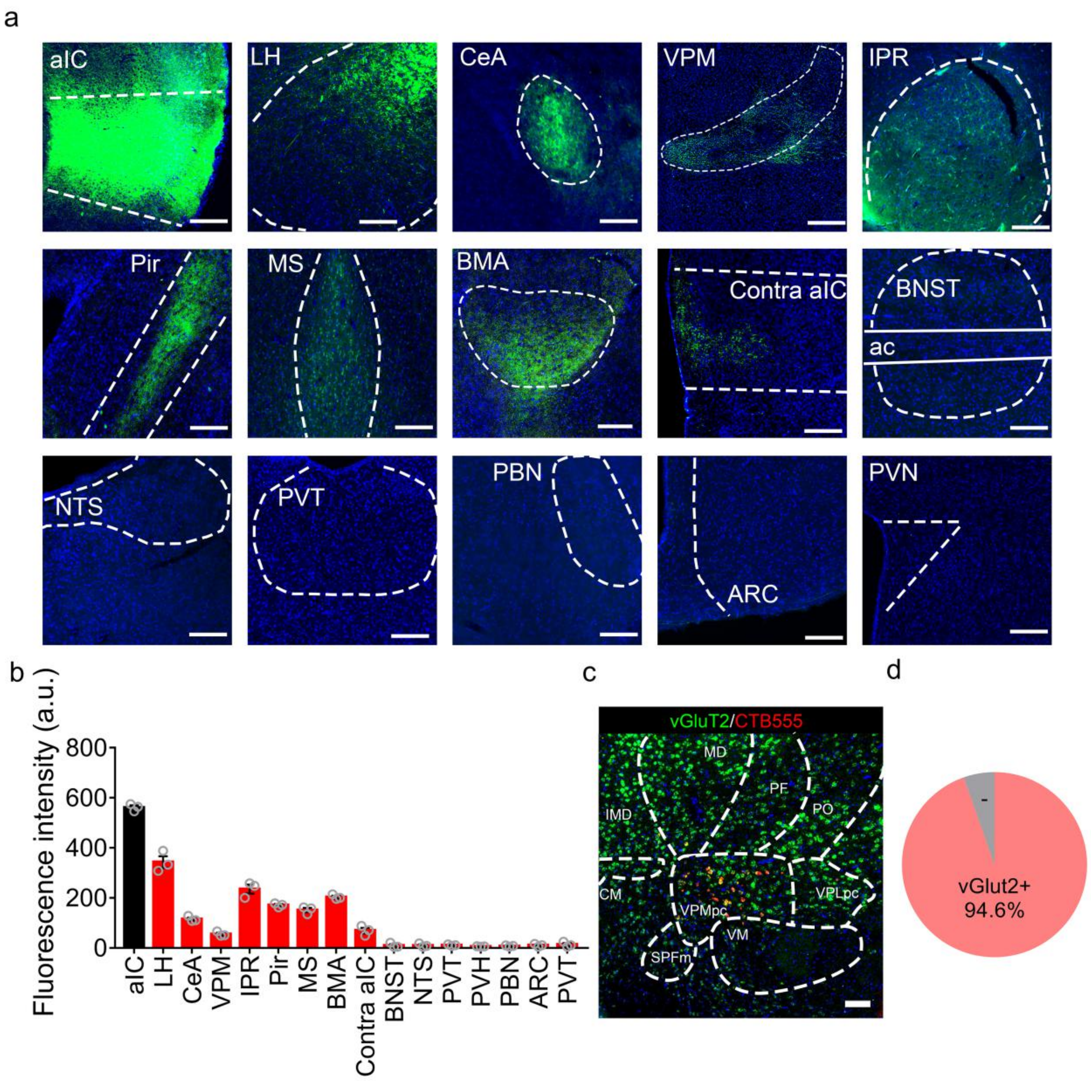

Supplementary Figure 10. Outputs of the aIC.

(a,b) AAV-hSyn-DIO-eGFP injected into the right aIC of *Camk2a-Cre* mice outputs throughout the brain (a) and positive axons were quantified (b). CeA, central amygdaloid area; IPR, interpeduncular nucleus rostral subnucleus; Pir, piriform cortex; VPM, ventral posteromedial thalamic nucleus; BMA, basomedial amygdaloid nucleus, anterior part; MS, medial septal nucleus; PVH, paraventricular thalamic nucleus; BNST, bed nucleus of the stria terminalis; ac, anterior commissure; PVT, paraventricular thalamic nucleus. Scale bar, 250 µm. Data are presented as means ± SEM. (c) Representative image from the area VPMpc showing the CTB555 labeled-cell bodies in red and *Slc17a6* mRNA positive cell nuclei in green 7 days after injection of CTB555 in the right aIC. Scale bar, 250 µm. MD, Mediodorsal nucleus of thalamus; IMD, Intermediodorsal nucleus of the thalamus; PF, Parafasciular nucleus; PO,

Posterior complex of the thalamus; VPLpc, Ventral posterolateral nucleus of the thalamus, parvicellular part; VM, Ventral medial nucleus of the thalamus; SPFm, Subparafasciular nucleus, magnocellular part; CM, Central medial nucleus of the thalamus. (d) Percentage of CTB555 transported from aIC neurons colocalized with vGluT2+ neurons in the VPMpc was quantified (n = 5 sections from 3 mice). Source data are provided as a Source Data file.

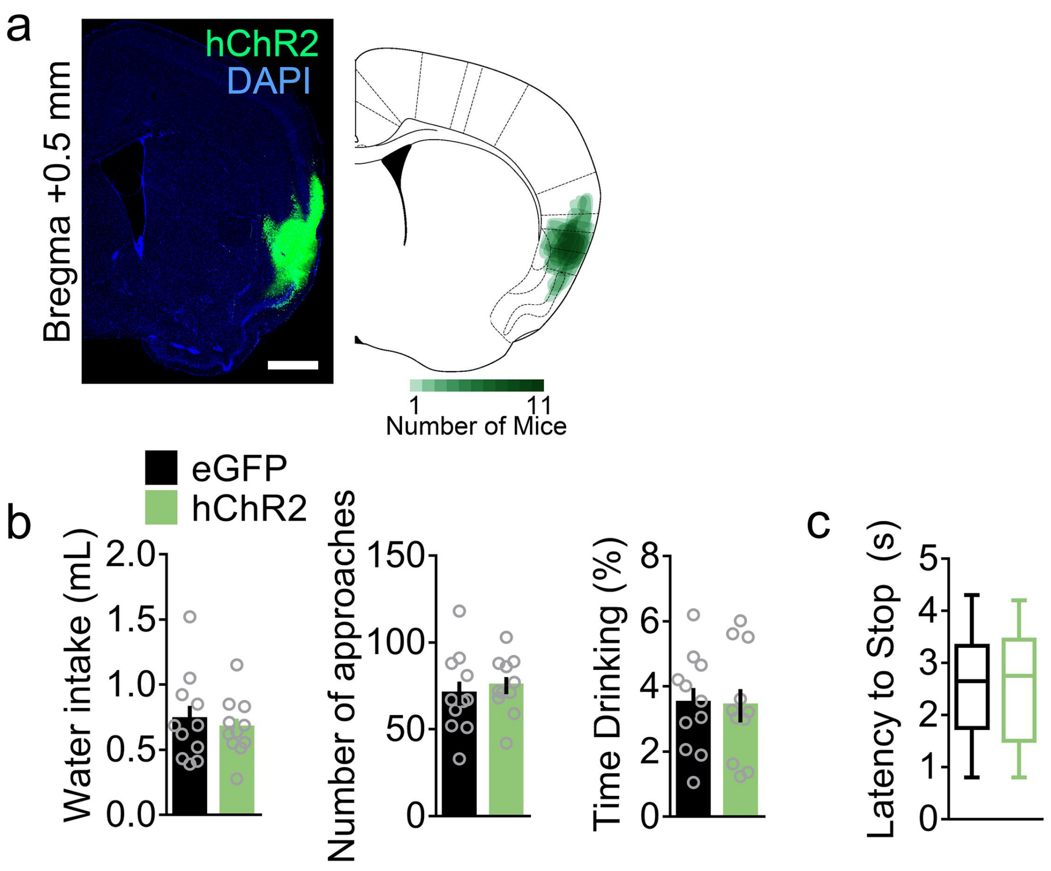

Supplementary Figure 11. Activation of right aIC^{CamKII}-to-LH projection terminals has no effect on drinking or mating behavior.

(a) Sample slice from mice with DIO-hChR2-eGFP virus injected into the right aIC in *Camk2a-Cre* mice. Blue, DAPI; green, viral targeting. Dashed black lines are boundaries of subregions. Overlay of DIO-hChR2-eGFP expression in 11 mice. Scale bar, 1 mm. (b) Total water consumption (left), number of licks (middle) and total drinking time (right) were measured (n = 11 mice per group). (c) Quantification of latency to stop mating (n = 6 for eGFP mice, n = 7 for hChR2 mice). Two-tailed unpaired t test. Data are presented as means ± SEM. Source data are provided as a Source Data file.

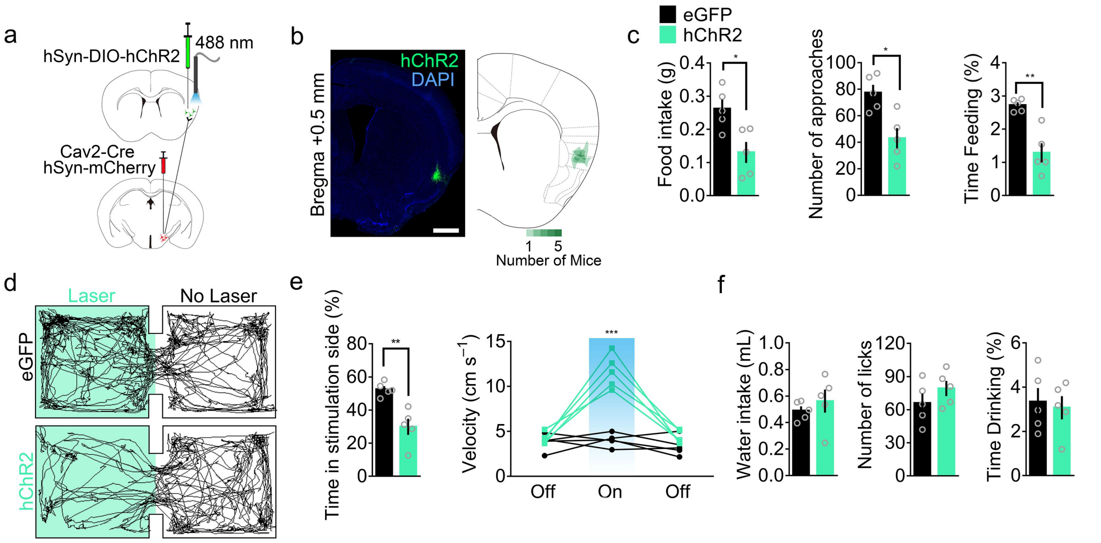

Supplementary Figure 12. Activation of the right aIC^{CamKII} neurons that project to LH reduces feeding behavior, but not drinking or mating behaviors.

(a) The image shows injection of Cav2-Cre into the right LH and hChR2-eGFP into the right aIC and fiber optic implanted above the right aIC in wild-type mice. (b) Sample slice from the wild-type mice with DIO-hChR2-eGFP virus injected into the right aIC and Cav2-Cre virus expressed in the LH. Blue, DAPI; green, viral targeting. Dashed black lines are boundaries of subregions. Overlay of DIO-hChR2-eGFP expression in 5 mice. Scale bar, 1 mm. (c) Total food consumption (left), number of approaches to food (middle) and the percentage of feeding time (right) were measured (n = 5 mice per group). (d) Representative locomotor trace of a mouse with the right aIC neurons that project to LH expressing eGFP or hChR2 and receiving 20 Hz photostimulation in the laser compartment. (e) Percentage of time spent (left) and locomotor activity (right) in laser or no laser sides (n = 5 mice per group). (f) Total water consumption (left), number of licks (middle) and total drinking time (right) were measured (n = 5 mice per group). Two-tailed unpaired t test. $*P < 0.05$; $**P < 0.01$. Data are presented as means ± SEM. Source data are provided as a Source Data file.

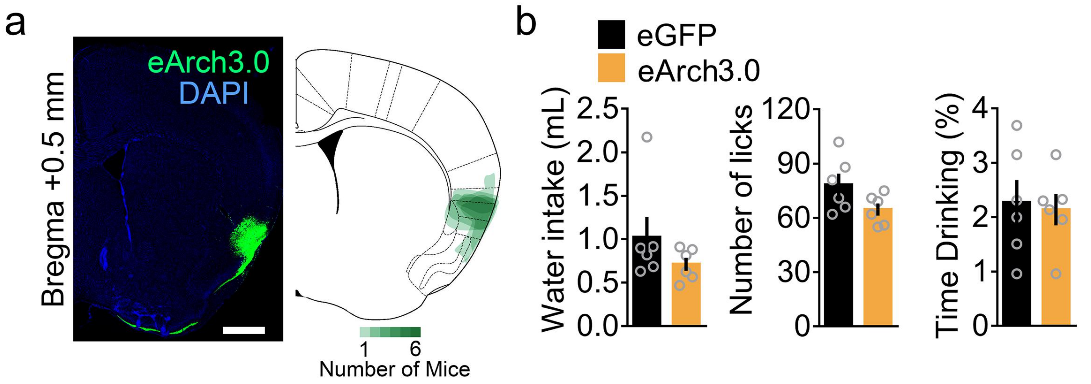

Supplementary Figure 13. Inhibition of the right aIC^{CaMKII}-to-LH projection terminals has no effect on drinking behaviors.

(a) Sample slice from mice with DIO-eArch3.0-eGFP virus injected into the right aIC in *Camk2a-Cre* mice. Blue, DAPI; green, viral targeting. Dashed black lines are boundaries of subregions. Overlay of DIO-hChR2-eGFP expression in 6 mice. Scale bar, 1mm. (b) Inhibition of the right aIC^{CaMKII}-to-LH projection terminals has no effect on total water consumption (left), number of licks (middle) and total drinking time (right) ($n = 6$ mice per group, Two-tailed unpaired t test). Data are presented as means ± SEM. Source data are provided as a Source Data file.
