# Supplementary material for: The anterior insular cortex unilaterally controls feeding in response to aversive visceral stimuli in mice
Source: Nat Commun. 2020 Jan 31;11:640. doi: 10.1038/s41467-020-14281-5 (PMC6994462; doi:10.1038/s41467-020-14281-5)
Supplement: Supplementary file 3 — Reporting Summary [file 41467_2020_14281_MOESM3_ESM.pdf]

nature research

Corresponding author(s): Shuang Qiu

Last updated by author(s): Nov 19, 2019

# Reporting Summary

Nature Research wishes to improve the reproducibility of the work that we publish. This form provides structure for consistency and transparency in reporting. For further information on Nature Research policies, see Authors & Referees and the Editorial Policy Checklist.

## Statistics

For all statistical analyses, confirm that the following items are present in the figure legend, table legend, main text, or Methods section.

| n/a | Confirmed | |
|---|---|---|
| ☐ | ☒ | The exact sample size (*n*) for each experimental group/condition, given as a discrete number and unit of measurement |
| ☐ | ☒ | A statement on whether measurements were taken from distinct samples or whether the same sample was measured repeatedly |
| ☐ | ☒ | The statistical test(s) used AND whether they are one- or two-sided<br>*Only common tests should be described solely by name; describe more complex techniques in the Methods section.* |
| ☒ | ☐ | A description of all covariates tested |
| ☐ | ☒ | A description of any assumptions or corrections, such as tests of normality and adjustment for multiple comparisons |
| ☐ | ☒ | A full description of the statistical parameters including central tendency (e.g. means) or other basic estimates (e.g. regression coefficient) AND variation (e.g. standard deviation) or associated estimates of uncertainty (e.g. confidence intervals) |
| ☐ | ☒ | For null hypothesis testing, the test statistic (e.g. *F*, *t*, *r*) with confidence intervals, effect sizes, degrees of freedom and *P* value noted<br>*Give P values as exact values whenever suitable.* |
| ☒ | ☐ | For Bayesian analysis, information on the choice of priors and Markov chain Monte Carlo settings |
| ☒ | ☐ | For hierarchical and complex designs, identification of the appropriate level for tests and full reporting of outcomes |
| ☒ | ☐ | Estimates of effect sizes (e.g. Cohen's *d*, Pearson's *r*), indicating how they were calculated |

*Our web collection on statistics for biologists contains articles on many of the points above.*

## Software and code

Policy information about availability of computer code

| | |
|---|---|
| Data collection | MATLAB (R2017b), MultiClamp (v.700b), pClamp(v.10), CellSens(v.2.2), FV10-ASW Viwer (Ver 4.2b), OlyVIA (Ver 2.9), Smart (Ver 3.0) |
| Data analysis | MATLAB (R2017b), ImageJ (v.1.48), Clampfit (v.10.4), Graphpad Prism 6. |

For manuscripts utilizing custom algorithms or software that are central to the research but not yet described in published literature, software must be made available to editors/reviewers. We strongly encourage code deposition in a community repository (e.g. GitHub). See the Nature Research guidelines for submitting code & software for further information.

## Data

Policy information about availability of data

All manuscripts must include a data availability statement. This statement should provide the following information, where applicable:

- Accession codes, unique identifiers, or web links for publicly available datasets
- A list of figures that have associated raw data
- A description of any restrictions on data availability

Data available on request from the authors

# Field-specific reporting

Please select the one below that is the best fit for your research. If you are not sure, read the appropriate sections before making your selection.

☒ Life sciences ☐ Behavioural & social sciences ☐ Ecological, evolutionary & environmental sciences

For a reference copy of the document with all sections, see nature.com/documents/nr-reporting-summary-flat.pdf

# Life sciences study design

All studies must disclose on these points even when the disclosure is negative.

| | |
|---|---|
| Sample size | No statistical methods were used to pre-determine sample sizes, but our sample sizes are similar to those reported in previous studies. |
| Data exclusions | We excluded those mice that were subsequently found that the placement of the opto-fibers was misplaced or there was no AAV expression, or AAV expression was in the wrong place. |
| Replication | Multiple mice were used for all the experiments and observations of similar results across mice were used to infer replication. All attempts at replication were successful. |
| Randomization | Mice were assigned randomly to the experimental and control groups. Experimental treatments were also randomized. |
| Blinding | For c-Fos staining and behavioral test, data collection and analysis were performed blindly. |

# Reporting for specific materials, systems and methods

We require information from authors about some types of materials, experimental systems and methods used in many studies. Here, indicate whether each material, system or method listed is relevant to your study. If you are not sure if a list item applies to your research, read the appropriate section before selecting a response.

## Materials & experimental systems

| n/a | Involved in the study | |
|---|---|---|
| ☐ | ☒ | Antibodies |
| ☒ | ☐ | Eukaryotic cell lines |
| ☒ | ☐ | Palaeontology |
| ☐ | ☒ | Animals and other organisms |
| ☒ | ☐ | Human research participants |
| ☒ | ☐ | Clinical data |

## Methods

| n/a | Involved in the study | |
|---|---|---|
| ☒ | ☐ | ChIP-seq |
| ☒ | ☐ | Flow cytometry |
| ☒ | ☐ | MRI-based neuroimaging |

## Antibodies

**Antibodies used**

Primary antibodies:
Rabbit anti-C-Fos (226 003, SYSY System), dilution 1:10000
Mouse anti-CaMKII(ab22609, Abcam), dilution 1:500
Mouse anti-GAD67 (MAB5406 EMD Millipore), dilution 1:1000
Secondary antibodies
Goat anti-rabbit Alexa Fluor 488 (a11008, Life Technologies), dilution 1:2000
Donkey anti-rabbit Alexa Fluor 555 (a10040, Life Technologies), dilution 1:2000
Goat anti-mouse Alexa Fluor 488 (a11029, Life Technologies), dilution 1:2000

**Validation**

Rabbit anti-C-Fos (226 003, SYSY System)
Adamsky A, Kol A, Kreisel T, Doron A, Ozeri-Engelhard N, Melcer T, Refaeli R, Horn H, Regev L, Groysman M, London M, et al. Astrocytic Activation Generates De Novo Neuronal Potentiation and Memory Enhancement. Cell 2018: Jun 28;174(1):59-71.

Mouse anti-CaMKII(ab22609, Abcam)
Dheeraj S. Roy, Takashi Kitamura, Teruhiro Okuyama, Sachie K. Ogawa, Chen Sun, Yuichi Obata, Atsushi Yoshiki, and Susumu Tonegawa et al. Distinct Neural Circuits for the Formation and Retrieval of Episodic Memories. Cell 2017 Aug 24;170(5):1000-1012.

Mouse anti-GAD67 (MAB5406 EMD Millipore)
Validated in J Dimidschstein, Q Chen, R Tremblay, SL Rogers, GA Saldi, et. al. A viral strategy for targeting and manipulating interneurons across vertebrate species. Nature Neuroscience 2016 Dec; 19(2): 1743-1749.

## Animals and other organisms

Policy information about studies involving animals; ARRIVE guidelines recommended for reporting animal research

**Laboratory animals**

We use C57BL/6 mice, CamKII-Cre mice (B6.Cg-Tg(Camk2a-cre)T29-1Stl/J, strain 005359, RRID:IMSR_JAX:005359) and vGluT2-ires-Cre mice (B6J.129S6(FVB)-Slc17a6tm2(cre)Lowl/MwarJ, strain 028863, RRID:IMSR_JAX:028863) in our experiments (6-12 weeks).

**Wild animals**

The study did not involve wild animals.

| | |
|---|---|
| Field-collected samples | This study does not include field-collected samples. |
| Ethics oversight | Animal experiments were conduct in accordance with the Guidelines for the Care and Use of Laboratory Animals of Zhejiang University. |

Note that full information on the approval of the study protocol must also be provided in the manuscript.
